# Daily Profiles of Neuropeptides, Catecholamines, and Neurotransmitter Receptors in the Chicken Pineal Gland

**DOI:** 10.3389/fphys.2018.01972

**Published:** 2019-01-15

**Authors:** Iwona Adamska, Monika Malz, Bogdan Lewczuk, Natalia Blügental, Magdalena Aleksandra Markowska, Robert Meronka, Paweł Marek Majewski

**Affiliations:** ^1^Department of Animal Physiology, Faculty of Biology, University of Warsaw, Warsaw, Poland; ^2^Department of Histology and Embryology, Faculty of Veterinary Medicine, University of Warmia and Mazury in Olsztyn, Olsztyn, Poland; ^3^Department of Ecology, Faculty of Biology, University of Warsaw, Warsaw, Poland

**Keywords:** pineal gland, circadian rhythm, chicken, neurotransmitters, catecholamines, VIP, PACAP, NA

## Abstract

The avian pineal gland is one of three central biological clocks that contain all the components of a circadian system: a photoreceptive input, oscillator, and rhythmically secreted melatonin (MEL) as an effector. The biosynthesis of MEL is regulated by the neurotransmitters noradrenaline (NA), vasoactive intestinal peptide (VIP), and pituitary adenylate cyclase-activating polypeptide (PACAP). The aim of the present study was to characterize the daily profile of neurotransmitters and their receptors in the pineal gland of male Hy-Line chickens housed under controlled light (12:12 light:dark) conditions. The pineal glands were isolated from 16-day-old birds every 2 h over a 24-h period, immediately after decapitation. The catecholamine content was measured using HPLC with electrochemical detection, whereas expression of VIP and PACAP was measured using quantitative real-time PCR (RT-qPCR) assays and Western blotting. Expression of the neurotransmitter receptors was also measured using RT-qPCR. We found daily changes in NA content, with elevated nocturnal levels, whereas the NA receptor was expressed in antiphase. Although we did not observe daily changes in VIP and PACAP protein levels, we found prominent diurnal changes in the expression of the *Vip* and *Pacap* genes. We also detected precursors of NA, 3,4-dihydroxy-L-phenylalanine (DOPA), and dopamine (DA) in the pineal glands, in addition to the DA metabolites. Our results provide the first evidence that the pineal gland itself may synthetize the neurotransmitters needed to regulate MEL biosynthesis.

## Introduction

Daily changes in biological parameters are essential properties of virtually all living organisms studied to date (Dunlap et al., 1999; Golden and Canales, 2003; Schibler, 2005). These daily changes in physiological state, called circadian rhythms, are generated by endogenous oscillators that are active for almost 24 h, even when organisms are placed in unchanging environments such as constant darkness (dark–dark: D:D). Endogenous oscillators are in turn synchronized to the local time via the detection of ambient cues, so that the endogenous phase corresponds reliably with an environmental phase. The dominant ambient cues for most species is the light-dark (L:D) cycle, and specialized photoreceptive and phototransduction mechanisms have evolved in biological clock systems. The biological clock consists of an input-pacemaker-output system. In vertebrates, the centers regulating the circadian rhythm reside within specialized structures. In mammals, the central biological clock is located in the hypothalamic suprachiasmatic nuclei (SCN) (Cassone, 1998). In contrast, the central biological clock in birds consists of three independent oscillators: the SCN, the retina of eyes, and pineal gland, with the pineal gland as the conductor (Zimmerman and Menaker, 1979; McGoogan and Cassone, 1999; Yoshimura et al., 2000). Moreover, in birds each central clock has the whole input-pacemaker-output system. Interestingly, each clock may interact with the other clocks and together they form one functional unit called the central clocking system (CCS) (Kumar et al., 2004). In the past, many studies have been carried out to check the degree of autonomy of individual clocks in the absence of other oscillators. These investigations revealed that the degree of clocks autonomy varies between species. It was demonstrated that the pineal gland is the key oscillator in passerine birds (Gwinner, 1978; Zimmerman and Menaker, 1979; Ebihara and Kawamura, 1981), whereas the retina is crucial in galliform and columbiform birds (Ebihara et al., 1984; Underwood, 1994). The pineal gland indoleamine hormone, melatonin (MEL), is synthesized rhythmically throughout the L:D cycle, with a nocturnal peak and a diurnal basal level. Melatonin is immediately released into the peripheral circulation and, therefore, an increase in the blood levels of MEL corresponds with the nocturnal period. Consequently, MEL conveys information about external light and the activity state of the central oscillator (Barclay et al., 2012).

Melatonin biosynthesis in both mammals and birds is regulated by sympathetic stimulation of the pineal gland arising from the superior cervical ganglia, which in turn receive input from SCN (Pratt and Takahashi, 1987). Sympathetic terminals release noradrenaline (NA), which in birds acts exclusively through α_2_-adrenergic receptors. Activation of α_2_-adrenergic G protein-coupled receptors, in birds encoded by *Alfa2A* gene, is associated with inhibition of cyclic adenosine monophosphate (cAMP), which in turn leads to reduced MEL biosynthesis (Pratt and Takahashi, 1987).

Two additional neurotransmitters crucial for the regulation of MEL biosynthesis are vasoactive intestinal peptide (VIP) and pituitary adenylate cyclase-activating polypeptide (PACAP). VIP and PACAP belong to the secretin peptide family and are highly conserved across species (Laburthe and Couvineau, 2002; Dickson and Finlayson, 2009). Both neuropeptides act through activation of two common G protein-coupled receptors, VPAC1 and VPAC2; additionally, PACAP also acts via its specific receptor PAC1, another membrane-associated protein which shares significant homology with members of the G protein-coupled class B glucagon/secretin receptor family (Laburthe and Couvineau, 2002). Ligand activation of VPAC1, VPAC2, and PAC1 receptors leads mainly to stimulation of adenylate cyclase (AC) and a consequent increase in cAMP level; however, stimulation of other intracellular messengers such as phospholipase D or calcium has also been observed (Dickson and Finlayson, 2009).

Although MEL biosynthesis is known to be regulated by the three described neurotransmitters, other neurotransmitters may also be involved in this process. In the rat, for example, dopamine (DA) may also be involved in the regulation of MEL biosynthesis (Gonzalez et al., 2012). Furthermore, various catecholamines have also been detected in duck pineal glands (Lewczuk et al., 2014).

Based on these observations, the aims of this study were to determine the content and daily profiles of NA and its precursors, DOPA and DA, as well as the DA metabolites 3,4-dihydroxyphenylacetic acid (DOPAC) and homovanillic acid (HVA), in the pineal glands of 16-day-old male chickens. Additionally, we also investigated the expression of VIP, PACAP, and neurotransmitter receptors.

## Materials and Methods

### Animals and Experimental Design

Experiments were performed on 16-day-old male Hy-Line chickens (*Gallus gallus domesticus* L.) kept under controlled conditions from the day of hatch. The chicks were transported from a commercial hatchery to the animal facility of the Faculty of Biology, University of Warsaw, on the day of hatch and kept under a strictly controlled 12 h:12 h L:D cycle using strip lighting with an intensity of 250 lux; the light came on at 6:00 am (Zeitgeber Time 0, ZT 0). The temperature was 32 ± 2°C during the first week and was then gradually decreased to 24 ± 2°C. The birds had *ad libitum* access to standard food and water.

All procedures were performed in accordance with the regulations of the Polish Ethical Council for the care and use of laboratory animals, and the European Community Directive for the ethical use of experimental animals. The protocol was approved by the First Local Ethical Council in Warsaw (Permit No. 227/2011).

### Content Analysis of Catecholamines and Their Metabolites

#### Sample Preparation for the Catecholamine Assay

Six 16-day-old chickens were sacrificed every 2 h over a 24-h period, starting from ZT 2. The pineal glands were isolated under strip lighting with an intensity of 250 lux during the day and under dim red light with an intensity <10 lux at night, according to previously described protocol (Adamska et al., 2016a,b), immediately frozen in liquid nitrogen and stored at -80°C prior to further analysis. The experiments were performed twice.

The frozen pineal glands were sonicated (5 × 2 s, 1 W) in 100 μL of ice cold 0.1 M perchloric acid using a Vibra-Cell VC 70 ultrasonic processor equipped with a 2 mm probe (Sonics & Materials Inc., Newtown, CT, United States). The homogenate was incubated for 20 min in an ice bath and then centrifuged (15 min, 60000 ×*g*, 4°C). The supernatant was carefully transferred into an autosampler vial and the pellet frozen at -75°C for the protein assay. The catecholamine assay was completed within 4 h of sample preparation.

#### Catecholamine Assay

Catecholamine and metabolite content were measured using a chromatographic system composed of an LPG 3400M four-channel pump with a built-in degasser (Dionex, Sunnyvale, CA, United States), WPS 3000SL autosampler (Dionex, Sunnyvale, CA, United States), and CoulArray 5600A electrochemical detector equipped with two four-channel 6210 coulometric cells (ESA Inc., Chelmsford, MA, United States). The system was controlled by Chromeleon 6.8 (Dionex, Sunnyvale, CA, United States) and CoulArray 3.10 Data Station (ESA Inc., Chelmsford, MA, United States) software. Standards or samples were both injected at 20-μL volumes into the MG 150 mm × 3.2 mm i.d. column, which was filled with 3 μm C18 particles (ESA, Inc., Chelmsford, MA, United States). The column and coulometric cells were kept at 25°C. The mobile phase consisted of acetonitrile and a buffer containing 90 mM sodium phosphate dihydrate, 50 mM citric acid, 1.7 mM 1-octanesulfonic acid sodium salt, and 50 μM disodium EDTA (pH 3.05 with phosphoric acid), mixed in a ratio of 6:94 (*v*/*v*). The mobile phase was pumped at a flow rate of 0.5 mL/min. The potentials applied on successive electrodes were -150, 200, 350, and 450 mV. Data acquisition and integration of chromatograms were computed using CoulArray 3.10 Data Station software. The amperage on a 200 mV electrode was used to quantify DOPA, DOPAC, DA, and NA content, whereas the amperage on a 350 mV electrode was used to measure HVA.

#### Protein Assay

After pineal homogenate centrifugation, the pellet was dissolved in 750 μL of a 1 M sodium hydroxide solution. The obtained solution was diluted 1:1 with water and used to determine the protein content using the Bradford microplate assay. Solutions of bovine serum albumin in 0.5 M sodium hydroxide served as standards to prepare the calibration curve.

### Analysis of the Expression of Neuropeptides and Their Receptors

#### mRNA Isolation and Quantification

Six 16-day-old chickens were sacrificed every 2 h over a 24-h period, starting from ZT 2. The pineal glands were isolated under strip lighting with an intensity of 250 lux during the day, and under dim red light with a light intensity of <10 lux at night, immediately frozen in liquid nitrogen and stored at -80°C prior to further analysis. The experiments were performed twice.

Total RNA was isolated from the pineal glands using the AxyPrep Multisource Total RNA Miniprep Kit (Axygen Biosciences, CA, United States) according to the manufacturer’s protocol. The concentration and quality of the mRNA were assessed using a spectrophotometer (BioPhotometer^®^ D30, Eppendorf, Germany). DNase treatment was performed with RQ1-RNase-Free DNase (Promega, WI, United States) following the instructions in standard manuals. The reaction mixtures for reverse transcription (RT) contained 1000 ng of total RNA, 2 μL of Maxima Enzyme Mix, 4 μL of 5× Maxima Reaction Mix (Maxima First Strand cDNA Synthesis Kit, Thermo Scientific, MA, United States), and nuclease-free water (NFH_2_O, Thermo Scientific, MA, United States), in a total reaction volume of 20 μL. RT reactions were performed in a thermal cycler (C1000 Touch, Bio-Rad, CA, United States) with the following conditions: 25°C for 10 min, 50°C for 30 min, and finally 85°C for 5 min. The RT products were used in quantitative real-time PCR (RT-qPCR) assays performed in 48-well transparent plates (MicroAmp Fast Optical 48-Well Reaction Plate, Applied Biosystems/Thermo Fisher Scientific, MA, United States). Each RT-qPCR mixture contained a cDNA template (10% of the RT product), 5 μL of 2× SYBR^TM^ green I PCR master mix (Kapa Sybr Fast Universal qPCR Kit, KapaBiosystem, MA, United States), 0.75 μM of gene-specific forward and reverse primers (Table 1), and NFH_2_O, in a total reaction volume to 12.5 μL. The reactions were performed in a thermal cycler (StepOne^TM^ Real Time PCR System, Applied Biosystems/Thermo Fisher Scientific, MA, United States) using the following conditions: 95°C for 20 s, followed by 40 cycles of denaturation (95°C for 3 s), annealing with extension (63°C for *Vip*, *Pacap* and *Tbp*, 62°C for *Alfa2A*, 61°C for *Vpac1* and *Vpac2*, and 58°C for *Pac1* genes; 30 s for each primer) and reamplification (95°C for 15 s, with a subsequent reduction of 0.3°C every cycle until 60°C). cDNA fragments of the *Vip*, *Pacap*, *Vpac1*, *Vpac2*, *Pac1*, *Alfa2A*, and *Tbp* (TATA-binding protein, reference gene) genes were purified and used as quantification standards (10^8^–10^1^) for singleplex qPCR standard curve method (absolute quantitation). Transcript-level quantification was performed using Applied Biosystems software: StepOnePlus Real Time PCR System, Version 2.2.2. Each sample was assayed in duplicate. The results were normalized to *Tbp* transcript levels and expressed as the number of mRNA copies per 100 copies of *Tbp* mRNA. Pulled cDNA with a well-known level of gene transcription was used as an internal control between plates. RNA isolation and RT-qPCR were carried out within 1 month of pineal gland collection.

**Table 1:** Primers used for RT-qPCR analysis.

Gene	Primer set
*Tbp*	**F** CAGACTCTTACCACAGCCCCTTT
	**R** CAAGTTTGCAACCAAGATTCACC
*Vip*	**F** CCATGGGTCCTTAAAGTCTGAA
	**R** TTTGGCTGGATTTAACTCTTCC
*Pacap*	**F** ATCTTCAGCAAAGCCTACAGGA
	**R** TGTTTATACCTTTTCCCCAGGA
*Alfa2A*	**F** CTTGCTCATGCTCTTCACCG
	**R** CGATGGCTTGTGTGATGGAC
*Vpac1*	**F** CCCTCTCTTTGGCATTCACTAC
	**R** GGTGGTGGTACTTCATGTCTGA
*Vpac2*	**F** ATTCAAGGGCTCAGTCATTCAT
	**R** GGGGCAGGTGTCTTTGTTATTA
*Pac1*	**F** TATTATGATGCCTGTGGCTTTG
	**R** TTGCACGTAGGATGAATGAAAC


#### Western Blot Analysis

Three 16-day-old chickens were sacrificed every 2 h over a 24-h period, starting from ZT 2. The pineal glands were isolated under strip lighting with an intensity of 250 lux during the day and under dim red light with an intensity of <10 lux at night, immediately frozen in liquid nitrogen and stored at -80°C prior to further analysis. The experiments were performed twice.

Levels of VIP and PACAP were analyzed by Western immunoblotting. The pineal glands were sonicated singly in 40 μL of ice-cold RIPA buffer with 4 μL of Complete protease inhibitor cocktail (Roche Diagnostics, Germany) and centrifuged (10 min, 17000 rpm, 4°C) to pellet cellular residues. The protein concentration in the supernatant was determined using a Pierce^TM^ BCA Protein Assay Kit (Thermo Fisher Scientific^TM^ Pierce Protein Biology, MA, United States) according to the manufacturer’s protocol. Solutions of proteins dissolved in Laemmli buffer (Bio-Rad, CA, United States) were denatured by boiling in a water bath for 5 min. The proteins were then resolved by gel electrophoresis (Bolt 4–12% Bis-Tris Plus, Invitrogen, Thermo Fisher Scientific, MA, United States; 50 μg protein per well) and electroblotted onto a PVDF membrane (Novex^®^ BLOT^TM^ Mini Blot Module, Thermo Fisher Scientific, MA, United States). The blots were blocked with 5% skimmed milk in TBST buffer for 1 h at room temperature and then washed with TBST buffer (5 min). Then they were incubated overnight at 4°C with primary antibodies (anti-VIP sc-7841 and anti-PACAP sc-7840, Santa Cruz Biotechnology, TX, United States) diluted 1:500 in TBST buffer containing 1% skimmed milk. Next, the blots were washed three times in TBST buffer and incubated for 1 h at room temperature with horseradish peroxidase-conjugated (sc-2768 Santa Cruz Biotechnology, TX, United States) secondary antibodies diluted 1:10 000 in TBST buffer containing 1% skimmed milk. The blots were then washed with TBST buffer (3 × 5 min). The blots were visualized by chemiluminescence detection with Pierce^®^ ECL Western Blotting Substrate (Thermo Scientific^TM^ Pierce^TM^ Protein Biology, MA, United States) on films (Medical X-Ray Film, AGFA HealthCare, Belgium) using a photographic developer and fixer (G138i Developer, G334 Rapid Fixer, AGFA HealthCare, Belgium) under dim red light. Thereafter, the blots were carefully washed with distilled water and incubated with 0.2 M sodium hydroxide for 5 min at room temperature to remove excess antibody from the blots. The blots were washed carefully again with distilled water and the steps described above were repeated with the same blots for β-actin, as a loading control, using anti-β-actin primary antibody (Sigma-Aldrich, MO, United States) diluted 1:10000 and secondary horseradish peroxidase-conjugated antibodies (sc-2004 Santa Cruz Biotechnology, TX, United States) diluted 1:10,000 dilution. Finally, all films were scanned (Epson Perfection 3200 Photo, Seiko Epson Corp., Japan) and optical density (OD) was analyzed in ImageJ and GraphPad Prism (GraphPad Software, CA, United States). The OD of the products was normalized to the β-actin OD.

### Statistical Analysis

The data presented as mean values ± SEM were compared using nonparametric statistical tests. Significance for daily changes in each parameter was assessed by the Kruskal–Wallis test, followed by Dunn’s Multiple Comparison *post hoc* test. The differences were considered significant at *p* < 0.05. Statistical analyses were performed using STATISTICA 10PL software (StatSoft/Dell, TX, United States).

## Results

### Pineal Catecholamines and Levels of Their Metabolites

**FIGURE 1:**
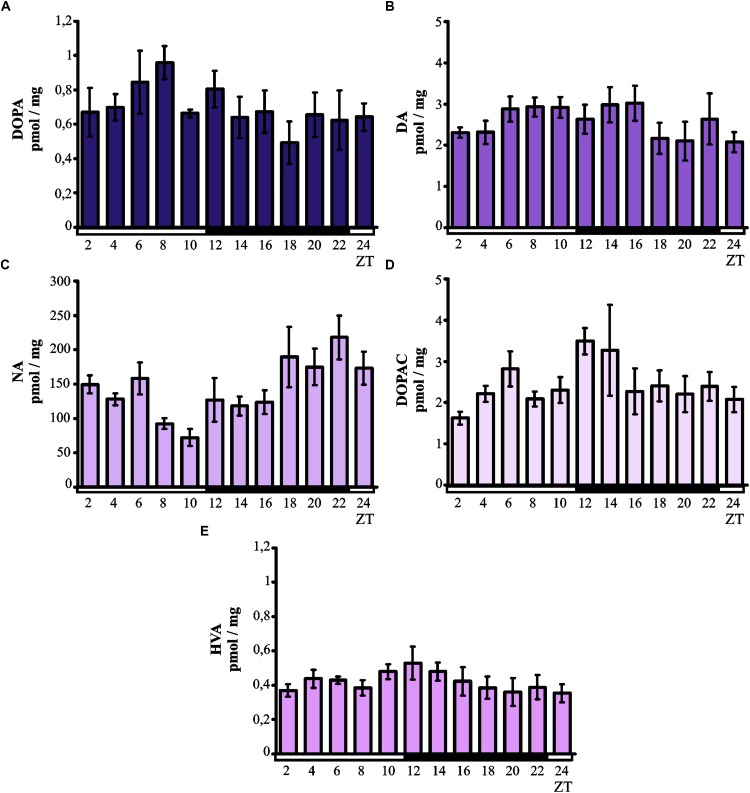
Daily changes in catecholamine and metabolite levels in the chicken pineal gland *in vivo*. The content of DOPA **(A)**, DA **(B)**, NA **(C)**, DOPAC **(D)**, and HVA **(E)** in 16-day-old chicken pineal glands were examined at 2-h intervals over a daily cycle. The bars represent the mean values ± SEM (*n* = 6).

Kruskal–Wallis test did not indicate significant daily variability in the level of DOPA in the chicken pineal glands (Figure 1A). However, although the DOPA levels remained low at all time points, there was a tendency for the levels to increase in the middle of the light phase. As with DOPA, Kruskal–Wallis test also did not reveal significant daily variability in the pineal level of DA (Figure 1B), which remained at a constant low level at all time points.

Kruskal–Wallis test indicated significant daily variability in the content of NA (*p* < 0.005; Figure 1C) in the chicken pineal glands. The NA levels were lower during the light phase than during the darkness, and Dunn’s multiple-comparisons *post hoc* test revealed significant difference between minimum (ZT 10) and maximum (ZT 22) values (*p* < 0.01).

Kruskal–Wallis test did not show significant daily variability in the pineal level of DOPAC (Figure 1D). However, although DOPAC content generally remained at a constant low level at all time points, there was a tendency for the levels to increase at the beginning of the dark period. Similarly, no significant daily variability in pineal HVA levels was revealed by Kruskal–Wallis test (Figure 1E). The content of this major catecholamine metabolite remained at a constant low level at all time points.

### Expression of Neuropeptides, Their Receptors, and the Alfa2A Receptor

#### Pineal Levels of *Vip* and *Pacap* mRNA

**FIGURE 2:**
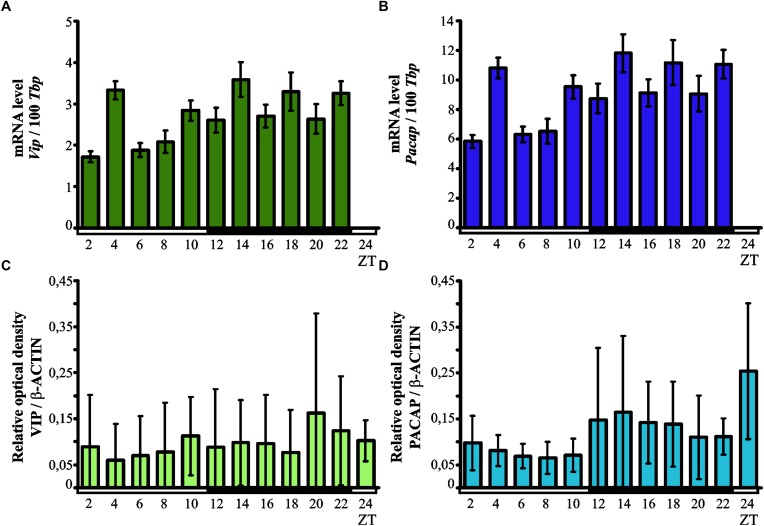
Daily changes in the expression of VIP and PACAP neurotransmitters. mRNA (**A,B**; *n* = 12) and protein levels (**C,D**; *n* = 3) in the chicken pineal glands *in vivo*. Genes expression in 16-day-old chicken pineal glands was examined at 2-h intervals over a daily cycle. The bars represent the mean values ± SEM.

Kruskal–Wallis test indicated significant daily variability in the expression of the *Vip* gene (*p* < 0.00001) in the chicken pineal gland (Figure 2A). Dunn’s multiple-comparisons *post hoc* test revealed that *Vip* mRNA levels were at their lowest at ZT 2 (*p* < 0.001), ZT 6 and ZT 8 (both *p* < 0.01), while the maximum level of expression was recorded in ZT4 (*p* < 0.01), ZT 14 (*p* < 0.05) and ZT 22 (*p* < 0.01).

Like with the *Vip* gene, Kruskal–Wallis test revealed that pineal *Pacap* transcription exhibited significant daily variability (*p* < 0.00001; Figure 2B). Similarly to the expression of *Vip* gene, Dunn’s multiple-comparisons *post hoc* test indicated that the lowest levels of *Pacap* mRNA were at ZT 2 (*p* < 0.001), ZT6 and ZT 8 (both *p* < 0.01), whereas the maximum transcript levels were found at ZT 4, ZT 14, and ZT 22 (all *p* < 0.01).

#### Pineal Levels of *Pac1*, *Vpac1*, *Vpac2*, and *Alfa2A* mRNA

**FIGURE 3:**
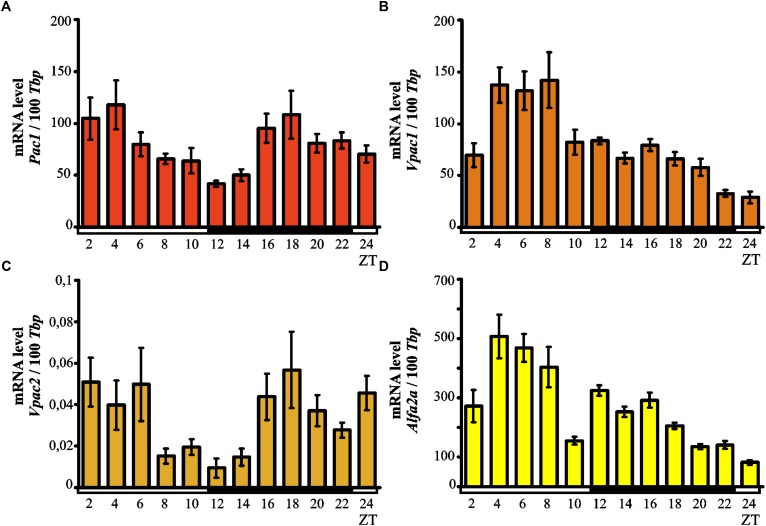
Daily changes in the mRNA levels of *Pac1* **(A)**, *Vpac1* **(B)**, *Vpac2* **(C)**, and *Alfa2A* **(D)** in the chicken pineal gland *in vivo*. The mRNA levels in 16-day-old chicken pineal glands were examined at 2-h intervals over a daily cycle. The bars represent the mean values ± SEM (*n* = 12).

Kruskal–Wallis test indicated significant daily variability in the pineal expression of the *Pac1* gene (*p* < 0.0007; Figure 3A). Dunn’s multiple-comparisons *post hoc* test did not show any significant differences in the levels of mRNA; however, we observed a tendency for high *Pac1* transcription values at the beginning of the light phase and the lowest transcript levels during transition from light to dark.

Kruskal–Wallis test showed significant daily variability in the pineal expression of the *Vpac1* gene (*p* < 0.00001; Figure 3B). Dunn’s multiple-comparisons *post hoc* test indicated that initially, at ZT 2, transcription levels were low while by ZT 4 the levels had increased twofold. The transcript levels of *Vpac1* remained high and reached a maximum at ZT 8 (*p* < 0.0000001); subsequently, *Vpac1* expression decreased rapidly at ZT 10 (*p* < 0.01) and persisted at a low level during the entire dark phase and beginning of the light phase. The lowest level of transcription was observed at ZT 24 (*p* < 0.01).

Kruskal–Wallis test revealed important daily variability in the pineal expression of the *Vpac2* gene (*p* < 0.0039; Figure 3C). Despite Dunn’s multiple-comparisons *post hoc* test not showing any significant differences, there was a tendency for *Vpac2* transcript levels to be at their lowest during transition from light to darkness.

Significant daily variability in pineal expression of *Alfa2A* was indicated by Kruskal–Wallis test (*p* < 0.00001; Figure 3D), which was confirmed by Dunn’s multiple-comparisons *post hoc* test. At ZT 2, the level of expression exhibited low values, rapidly increasing thereafter and reaching the maximum at ZT 4 (*p* < 0.01). Furthermore, the transcription persisted at a similar high level up to ZT 8 and then rapidly decreased at ZT 10 (*p* < 0.01). At the beginning of the dark period (ZT 12), the expression increased and then gradually decreased up to ZT 24 (*p* < 0.01), when it reached its minimal value.

#### Pineal VIP and PACAP Protein Levels

Kruskal–Wallis test did not indicate any significant daily variability in the pineal levels of both VIP and PACAP proteins (Figures 2C,D). The content of both neuropeptides remained low at all time points.

## Discussion

The data obtained in this study provide the first characterization of the daily profiles of almost all the neurotransmitters, as well as their receptors, that regulate MEL biosynthesis in the chicken pineal gland. Our results indicated a prominent daily rhythm of *Vip* and *Pacap* expression with markedly higher nocturnal values, whereas VIP and PACAP protein content showed no significant diurnal changes. The results at the protein level are in line with the those reported previously (Csernus et al., 2004), wherein an absence of daily fluctuations in PACAP content in the pineal glands of 6- and 10-week-old chickens kept under L:D 14:10 conditions was reported. It has also been shown that, in the chicken pineal gland, PACAP regulates the release of MEL but does not affect the rhythm of its synthesis (Nakahara et al., 2002). The results of studies carried out on mammals are contradictory. Moller et al. (1999) observed no daily changes in the pineal content of PACAP in young Wistar rats kept under L:D 12:12 conditions; in contrast, Fukuhara found significant daily changes in PACAP levels, with a nocturnal peak, in the pineal glands of 5-week-old Wistar rats also housed under L:D 12:12 conditions (Fukuhara et al., 1998).

Vasoactive intestinal peptide and PACAP are thought to be released only from nerve fibers, and the expression of the genes coding for these peptides in the pineal gland has not been studied using modern experimental approaches. Therefore, and to the best of our knowledge, our results from this study are the first to suggest that VIP and PACAP can be synthesized in the pineal glands.

Our results also indicated prominent daily changes in the expression of *Vpac1*, with a peak at ZT 8, signifying that the expression of the VIP and PACAP neurotransmitters and their VPAC1 receptor remained in antiphase. In contrast, the expression of *Vpac2* and *Pac1* did not show a pronounced diurnal rhythm. Moreover, the level of *Vpac2* was significantly lower than that of the other two receptors. Experiments carried out on Leghorn chickens have demonstrated that PACAP has a high affinity for the PAC1 receptor and lower affinity for the VPAC1 and VPAC2 receptors, whereas VIP has a high affinity for VPAC1 and VPAC2 (Zawilska et al., 2003). Binding between these neuropeptides and their receptors was found to be stable and reversible (Zawilska et al., 2003), similar to that observed in studies on young geese (Zawilska et al., 2004a) and turkeys (Zawilska et al., 2004b).

Our results further demonstrated that the NA content in the chicken pineal gland exhibited significant daily changes, with higher values recorded during the dark period. This result agrees with the results reported by Zawilska et al. (2005). Studies on 2-, 4-, 8-, 15-, 30-, and 57-day-old Leghorn chickens kept under L:D 12:12 conditions did not reveal any daily changes in the pineal NA content of the youngest chickens; however, in 30- and 57-day-old birds, the level of NA was higher and was elevated in the dark period (Zawilska et al., 2005), likely connected with the postembryonic development of the sympathetic innervation of the avian pineal glands (Sato, 2001). The differences between our results and those obtained by Zawilska et al. (2005) may also result from differences in the development of sympathetic innervation in different breeds of chickens. In contrast, Cassone and colleagues did not detect diurnal changes in the pineal NA content of 12-week-old chickens. Similarly, the level NA did not change during a 24-h period in the pineal glands of 14-week-old ducks (Lewczuk et al., 2014). The results of mammalian studies are contradictory and depend both on the species and length of the photoperiod. For example, in the pineal glands of Djungarian hamsters housed under L:D 14:10 conditions, the NA content remained constant during a 24-h period, whereas the NA content was elevated during the dark period under L:D 10:14 conditions (Miguez et al., 1996). Additionally, NA levels did not change during the day in Syrian hamsters, regardless of photoperiod conditions (Miguez et al., 1995; Moujir et al., 1997). In contrast, in the pineal glands of rats kept under a L:D 12:12 photoperiod, the level of NA was found to be elevated in the dark period (Miguez et al., 1998).

Our results have also shown the presence of DOPA, DA, DOPAC, and HVA in the chicken pineal gland, although we did not observe daily fluctuations in the level of these catecholamines and their metabolites. These results are partly in accordance with previously reported findings: Lewczuk et al. (2014) did not observed daily changes in the content of DA in the duck pineal gland, while Cassone et al. (1986) reported a small nocturnal peak of DA in the pineal gland of the chicken. The results of studies carried out on 14-week-old ducks indicated prominent daily changes in DOPA, DOPAC, and HVA content, with the highest values being observed in the dark period (Lewczuk et al., 2014). Rhythmical changes in the pineal content of DA and DOPAC, with higher nocturnal values, have also been reported in rodents (Miguez et al., 1995, 1996, 1998; Moujir et al., 1997).

Our results indicated a prominent daily rhythm in the expression of *Alfa2A*, a gene encoding for the α_2_-adrenergic receptor. Interestingly, this gene showed stronger diurnal expression, reaching a peak at ZT 4, indicating that *Alfa2A* expression remains in antiphase with the appearance of its ligand, NA. It has been demonstrated in chicken pinealocytes that NA inhibits the biosynthesis of MEL via α_2_-adrenergic receptors in a dose-dependent manner (Pratt and Takahashi, 1987). Zatz and Mullen (1988) cultured chicken pinealocytes for 5 days under light-red light (L:R) 12:12 conditions; subsequently, they were switched to constant red light (R:R), and incubated in medium containing 10^-5^ M NA for 4 h at 8 different time-points. Their results confirmed reports of the inhibitory effects of NA on MEL synthesis, which likely resulted from the increasing NA levels in the synaptic terminals of sympathetic nerves stimulating the expression of α_2_-adrenergic receptors in the pineal glands and consequently inhibiting MEL biosynthesis. Using non-physiological concentrations of NA in the medium, Zatz and Mullen achieved the same effect during the dark period. Additionally, rhythmical expression of α_1B_-adrenergic receptors with threefold higher nocturnal values was also observed in rat pineal glands. In these animals, expression of the α_1B_-adrenergic receptors was regulated by cAMP (Coon et al., 1997).

The results of our research indicated the presence of NA, its precursors DOPA and DA, as well as the DA metabolites DOPAC and HVA, in the chicken pineal gland. Although the function of all the listed compounds in the pineal gland is still not fully known, studies conducted on quails and zebra finches kept under L:D 14:10 conditions, as well as rats housed under a L:D 12:12 photoperiod, showed that DA binds to and activates α_2_-adrenergic receptors in various areas of the brain (Cornil and Ball, 2008), which has been suggested to initiate intracellular cascades different to those activated by NA (Zhang et al., 2004). Later studies in rats showed that rhythmic expression of D4, a gene encoding a DA receptor, occurs in the pineal glands (Kim et al., 2010). The D4 gene in the pineal gland is expressed at 100-fold higher levels than in other tissues, except the retina; interestingly, thyroid hormone was shown to be involved in the regulation of D4 expression (Kim et al., 2010). Recent studies have further shown that D4 receptors form heteromers with two different adrenergic receptors, α_1B_-D4 and β_1_-D4 (Gonzalez et al., 2012). The heteromers are formed in a circadian manner, and DA has been suggested to inhibit the biosynthesis and release of MEL via these heteromers (Gonzalez et al., 2012). However, it is still unclear whether the D4 receptors are also expressed in the avian pineal gland. Moreover, DA function in the regulation of MEL biosynthesis in birds has not been studied. The role of DOPA, a DA precursor, in the regulation of the MEL biosynthesis is also unknown; however, the results of studies conducted by Misu and coworkers suggested that DOPA itself fulfills all the criteria of a neurotransmitter (Misu et al., 2002).

Unlike mammals, the avian central biological clock consists of three independent oscillators: the suprachiasmatic nuclei, the retina of eyes, and the pineal gland. It has been shown that denervation of the pineal gland does not abolish the circadian rhythm of MEL biosynthesis in the chicken (Ralph et al., 1975). Isolated avian pineal glands or pinealocytes were also found to contain all the components of a circadian system: a photoreceptive input, oscillator, and rhythmically secreted MEL as an effector (Takahashi et al., 1984). This is possible due to the presence of pinopsin which is photoreceptive molecules. Pinopsin transmits a light signal to the oscillator where acts the transcription/translation-based feedback loop composed of positive and negative elements (Okano and Fukada, 2003). Pinopsin is synthesized in pinealocytes and the highest level of its transcript was found in the end of the light phase (Holthues et al., 2005). We suggest that other compounds regulating the pineal gland activity and MEL biosynthesis may also be synthetized by the avian pineal gland itself. The presence of catecholamine precursors, their metabolites, and mRNA of genes coding for protein neurotransmitters in the pineal glands supports the hypothesis that these compounds are not only transported to pinealocytes by sympathetic nerves, but are additionally synthesized directly in the pineal glands. Neurotransmitters are likely synthetized by pinealocytes or by pineal neurons, in agreement with the results of studies conducted on rats by Abreu et al. (1987). These authors demonstrated the circadian rhythm of tyrosine hydroxylase activity, an enzyme that converts L-tyrosine to L-DOPA in cells synthesizing catecholamines (Abreu et al., 1987). To verify this hypothesis, further *in vitro* studies on pinealocytes and pineal neurons are required.

## Author Contributions

IA planned, funded, designed, and coordinated the study, evaluated the results, and prepared the manuscript. MAM performed the neuropeptide experiments, analyzed the data, and critically revised the manuscript. BL performed the catecholamine experiments, analyzed the data, and critically revised the manuscript. NB performed the neuropeptide experiments and commented on the manuscript. MAM analyzed the western blotting data and commented on the manuscript. RM performed the statistical analysis and commented on the manuscript. PM planned the study, evaluated the results, and critically revised the manuscript.

## Conflict of Interest Statement

The authors declare that the research was conducted in the absence of any commercial or financial relationships that could be construed as a potential conflict of interest.
